# (η^6^-*p*-Cymene)bis­(trichlorido­stannyl)(triethoxy­phosphine-κ*P*)ruthenium(II)

**DOI:** 10.1107/S1600536809042986

**Published:** 2009-10-23

**Authors:** Sergey S. Shapovalov, Bruno Therrien

**Affiliations:** a Institut de Chimie, Université de Neuchâtel, Case postale 158, CH-2009 Neuchâtel, Switzerland

## Abstract

In the title complex, [RuSn_2_(C_10_H_14_)Cl_6_(C_6_H_15_O_3_P)], the Ru—Sn bond lengths [2.5619 (3) and 2.5669 (3) Å] are about 0.3 Å shorter than the sum of the covalent Ru and Sn radii (1.46 + 1.39 = 2.85 Å), in line with other structurally characterized arene ruthenium trichlorido­stannyl derivatives. The Ru(II) atom is surrounded by a *para*-cymene, a triethylphosphite and two trichloridostannyl ligands in a typical piano-stool coordination.

## Related literature

For the synthesis of the P(OMe)_3_ analogue (η^6^-*p*-cymene){bis­(trichlorido­stannyl-*κSn*)}(trimethyl­phosphite-*κP*)ruth­enium(II), see: Hodson & Simpson (2004 ▶). For the structures of other trichloro­stannyl arene ruthenium derivatives, see: Cordero *et al.* (2008 ▶); Korp & Bernal (1981 ▶); Alvarez *et al.* (1994 ▶); Therrien *et al.* (2009 ▶).
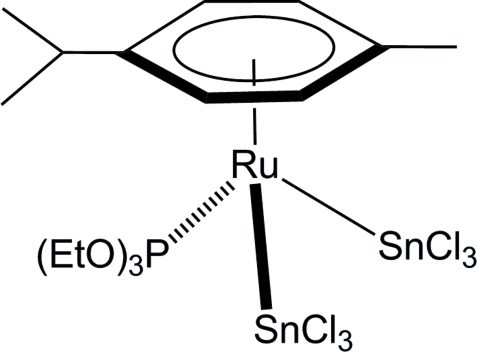


## Experimental

### 

#### Crystal data

[RuSn_2_(C_10_H_14_)Cl_6_(C_6_H_15_O_3_P)]
*M* *_r_* = 851.51
Orthorhombic, 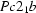

*a* = 8.8928 (4) Å
*b* = 16.2936 (6) Å
*c* = 18.9520 (11) Å
*V* = 2746.1 (2) Å^3^
*Z* = 4
Mo *K*α radiation
μ = 3.01 mm^−1^
*T* = 173 K
0.17 × 0.15 × 0.08 mm

#### Data collection

Stoe IPDS diffractometer
Absorption correction: refined from Δ*F* (Walker & Stuart, 1983 ▶) *T*_min_ = 0.616, *T*_max_ = 0.886
26284 measured reflections
4889 independent reflections
4558 reflections with *I* > 2σ(*I*)
*R*_int_ = 0.031

#### Refinement

*R*[*F* ^2^ > 2σ(*F* ^2^)] = 0.014
*wR*(*F* ^2^) = 0.024
*S* = 0.89
4889 reflections
268 parameters
1 restraint
H-atom parameters constrained
Δρ_max_ = 0.30 e Å^−3^
Δρ_min_ = −0.26 e Å^−3^
Absolute structure: Flack (1983 ▶), 2326 Friedel pairs
Flack parameter: −0.031 (11)

### 

Data collection: *EXPOSE* (Stoe, 2000 ▶); cell refinement: *CELL* (Stoe, 2000 ▶); data reduction: *INTEGRATE* (Stoe, 2000 ▶); program(s) used to solve structure: *SHELXS97* (Sheldrick, 2008 ▶); program(s) used to refine structure: *SHELXL97* (Sheldrick, 2008 ▶); molecular graphics: *ORTEP* (Farrugia, 1997 ▶); software used to prepare material for publication: *SHELXL97*.

## Supplementary Material

Crystal structure: contains datablocks global, I. DOI: 10.1107/S1600536809042986/dn2502sup1.cif

Structure factors: contains datablocks I. DOI: 10.1107/S1600536809042986/dn2502Isup2.hkl

Additional supplementary materials:  crystallographic information; 3D view; checkCIF report

## Figures and Tables

**Fig. 1.**
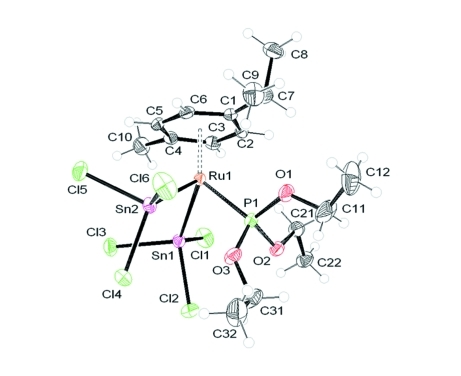
The molecular structure of (η6-p-cymene){bis(trichlorostannyl-κSn)}- (triethylphosphite-κP)ruthenium(II). Displacement ellipsoids are drawn at the 50% probability level.

## supplementary crystallographic information

### Comment

Insertion of tin dichloride into ruthenium-halogen bonds remains scarce, in spite of the rich chemistry of this metal. Recently, we reported the synthesis of neutral, anionic and cationic arene ruthenium complexes containing trichlorostannyl ligands (Therrien *et al.*, 2009). A strategy similar to the one used by Hodson & Simpson (Hodson & Simpson, 2004) to synthesize [(η^6^-^i^PrC_6_H_4_Me)Ru{P(OMe)_3_}(SnCl_3_)_2_] was employed. We have now synthesized the triethylphosphite analogue and obtained good quality crystals of the neutral complex [(η^6^-^i^PrC_6_H_4_Me)Ru{P(OEt)_3_}(SnCl_3_)_2_].

scheme 1 here

The single-crystal X-ray structure analysis of [(η^6^-^i^PrC_6_H_4_Me)Ru{P(OEt)_3_}(SnCl_3_)_2_] reveals a typical piano-stool geometry with the ruthenium atom being coordinated by a *para*-cymene, a triethylphosphite and two trichlorostannyl ligands, see Fig. 1. The Ru—Sn bond lengths [2.5619 (3) and 2.5669 (3) Å] are about 0.3 Å shorter than the sum of the covalent Ru and Sn radii (1.46 + 1.39 = 2.85 Å)(Cordero *et al.*, 2008), but are comparable to those found in other arene-Ru—Sn complexes (Korp & Bernal, 1981; Alvarez *et al.*, 1994; Therrien *et al.*, 2009). Similarly, the Ru—P bond distance [2.2579 (8) Å] is comparable to the one found in [(η^6^-^i^PrC_6_H_4_Me)Ru{P(OPh)_3_}Cl_2_] [2.2642 (8) Å] (Hodson & Simpson, 2004). The distance between Ru and the centroid of the arene ligand is normal at 1.779 Å. No meaningful interactions between independent complexes are observed in the crystal packing, the Cl···H-C distances ranging from 2.753 to 2.947 Å.

### Experimental

(η^6^-*p*-Cymene){bis(trichlorostannyl-*κSn*)} (triethylphosphite-*κP*)ruthenium(II), was disolved in hot chloroform, and crystals suitable for X-ray diffraction analysis were obtained, after days, by slow evaporation of the chloroform solution.

^1^H NMR (400 MHz, DMSO-d_6_, p.p.m.): 6.30 (d, 2H, H*_p_*_-cym_), 6.08 (d, 2H, H*_p_*_-cym_), 4.13 (br, 6H, CH_2_), 3.04 (sept, 1H, CH*_p_*_-cym_), 2.32 (s, 3H, CH_3_), 1.30 (m, 15H, CH_3_)

^13^C{^1^H} NMR (100 MHz, DMSO-d_6_, p.p.m.): 118.7 (C*_p_*_-cym_), 112.1 (C*_p_*_-cym_), 92.5 (CH*_p_*_-cym_), 91.8 (CH*_p_*_-cym_), 64.3 (CH_2_), 30.3 (CH), 23.2 (CH_3_), 19.5 (CH_3_), 16.1 (CH_3_)

^31^P NMR (162 MHz, DMSO-d_6_): 127.0 p.p.m. (t, *^2^J*_P—Sn_ = 358 Hz)

^119^Sn NMR (149 MHz, DMSO-d_6_): -241.4 p.p.m. (*d*)

### Refinement

The H atoms were included in calculated positions and treated as riding on their parent atoms, with C—H = 0.93–0.98 Å and with *U*_iso_(H) = 1.2 times *U*_eq_(C).

### Crystal data

**Table:**

[RuSn_2_(C_10_H_14_)Cl_6_(C_6_H_15_O_3_P)]	*F*(000) = 1640
*M**_r_* = 851.51	*D*_x_ = 2.060 Mg m^−^^3^
Orthorhombic, *P**c*2_1_*b*	Mo *K*α radiation, λ = 0.71073 Å
Hall symbol: P -2bc -2c	Cell parameters from 28214 reflections
*a* = 8.8928 (4) Å	θ = 1.3–25.6°
*b* = 16.2936 (6) Å	µ = 3.01 mm^−^^1^
*c* = 18.9520 (11) Å	*T* = 173 K
*V* = 2746.1 (2) Å^3^	Block, orange
*Z* = 4	0.17 × 0.15 × 0.08 mm

### Data collection

**Table:**

STOE IPDS diffractometer	4889 independent reflections
Radiation source: fine-focus sealed tube	4558 reflections with *I* > 2σ(*I*)
graphite	*R*_int_ = 0.031
Detector resolution: 0.81 pixels mm^-1^	θ_max_ = 25.2°, θ_min_ = 2.2°
φ oscillation scans	*h* = −10→10
Absorption correction: part of the refinement model (Δ*F*) Walker & Stuart, 1983	*k* = −19→19
*T*_min_ = 0.616, *T*_max_ = 0.886	*l* = −22→22
26284 measured reflections	

### Refinement

**Table:**

Refinement on *F*^2^	Secondary atom site location: difference Fourier map
Least-squares matrix: full	Hydrogen site location: inferred from neighbouring sites
*R*[*F*^2^ > 2σ(*F*^2^)] = 0.014	H-atom parameters constrained
*wR*(*F*^2^) = 0.024	*w* = 1/[σ^2^(*F*_o_^2^) + (0.0108*P*)^2^] where *P* = (*F*_o_^2^ + 2*F*_c_^2^)/3
*S* = 0.89	(Δ/σ)_max_ = 0.002
4889 reflections	Δρ_max_ = 0.30 e Å^−^^3^
268 parameters	Δρ_min_ = −0.26 e Å^−^^3^
1 restraint	Absolute structure: Flack (1983), 2326 Friedel pairs
Primary atom site location: structure-invariant direct methods	Flack parameter: −0.031 (11)

### Special details

**Table:**

Experimental. A crystal was mounted at 173 K on a Stoe Image Plate Diffraction System (Stoe & Cie, 2000) using Mo *K*α graphite monochromated radiation. Image plate distance 70 mm, φ oscillation scans 0 - 200°, step Δφ = 1.2°, 3 minutes per frame.
Geometry. All e.s.d.'s (except the e.s.d. in the dihedral angle between two l.s. planes) are estimated using the full covariance matrix. The cell e.s.d.'s are taken into account individually in the estimation of e.s.d.'s in distances, angles and torsion angles; correlations between e.s.d.'s in cell parameters are only used when they are defined by crystal symmetry. An approximate (isotropic) treatment of cell e.s.d.'s is used for estimating e.s.d.'s involving l.s. planes.
Refinement. Refinement of *F*^2^ against ALL reflections. The weighted *R*-factor *wR* and goodness of fit *S* are based on *F*^2^, conventional *R*-factors *R* are based on *F*, with *F* set to zero for negative *F*^2^. The threshold expression of *F*^2^ > σ(*F*^2^) is used only for calculating *R*-factors(gt) *etc*. and is not relevant to the choice of reflections for refinement. *R*-factors based on *F*^2^ are statistically about twice as large as those based on *F*, and *R*- factors based on ALL data will be even larger.

### Fractional atomic coordinates and isotropic or equivalent isotropic displacement parameters (Å^2^)

**Table:**

	*x*	*y*	*z*	*U*_iso_*/*U*_eq_	
C1	0.4369 (3)	0.18963 (16)	0.16104 (15)	0.0213 (6)	
C2	0.5192 (3)	0.15987 (16)	0.21998 (16)	0.0215 (7)	
H2	0.5780	0.1130	0.2148	0.026*	
C3	0.5144 (3)	0.19905 (16)	0.28568 (16)	0.0235 (7)	
H3	0.5705	0.1783	0.3230	0.028*	
C4	0.4245 (3)	0.27025 (15)	0.29587 (15)	0.0246 (6)	
C5	0.3507 (3)	0.30259 (17)	0.23696 (16)	0.0248 (7)	
H5	0.2959	0.3509	0.2416	0.030*	
C6	0.3579 (3)	0.26348 (17)	0.17084 (16)	0.0239 (7)	
H6	0.3089	0.2872	0.1326	0.029*	
C7	0.4264 (4)	0.13923 (18)	0.09405 (16)	0.0297 (7)	
H7	0.5221	0.1103	0.0878	0.036*	
C8	0.3037 (4)	0.0751 (2)	0.1061 (2)	0.0446 (9)	
H8A	0.3280	0.0429	0.1470	0.067*	
H8B	0.2969	0.0399	0.0656	0.067*	
H8C	0.2090	0.1021	0.1133	0.067*	
C9	0.3967 (4)	0.1888 (2)	0.02797 (17)	0.0429 (9)	
H9A	0.2987	0.2133	0.0308	0.064*	
H9B	0.4014	0.1535	−0.0125	0.064*	
H9C	0.4713	0.2311	0.0237	0.064*	
C10	0.4063 (4)	0.30860 (18)	0.36759 (16)	0.0366 (8)	
H10A	0.3943	0.3669	0.3626	0.055*	
H10B	0.4938	0.2974	0.3956	0.055*	
H10C	0.3191	0.2860	0.3903	0.055*	
C11	0.8662 (5)	0.1675 (3)	0.0268 (2)	0.0730 (15)	
H11A	0.9162	0.2135	0.0044	0.088*	
H11B	0.9420	0.1349	0.0507	0.088*	
C12	0.7944 (6)	0.1187 (3)	−0.0257 (2)	0.0658 (13)	
H12A	0.7593	0.0685	−0.0048	0.099*	
H12B	0.8648	0.1063	−0.0625	0.099*	
H12C	0.7107	0.1483	−0.0450	0.099*	
C21	0.9359 (3)	0.15199 (17)	0.21738 (17)	0.0309 (7)	
H21A	0.9312	0.1043	0.1869	0.037*	
H21B	0.8448	0.1535	0.2455	0.037*	
C22	1.0695 (3)	0.14577 (18)	0.26462 (17)	0.0325 (7)	
H22A	1.1587	0.1395	0.2366	0.049*	
H22B	1.0584	0.0991	0.2951	0.049*	
H22C	1.0774	0.1947	0.2926	0.049*	
C31	1.0134 (3)	0.3573 (3)	0.07419 (19)	0.0437 (8)	
H31A	1.0585	0.3066	0.0577	0.052*	
H31B	1.0763	0.3795	0.1114	0.052*	
C32	1.0025 (5)	0.4164 (2)	0.0157 (2)	0.0662 (13)	
H32A	0.9432	0.3932	−0.0216	0.099*	
H32B	1.1015	0.4286	−0.0016	0.099*	
H32C	0.9558	0.4659	0.0322	0.099*	
Cl1	0.84138 (10)	0.27207 (5)	0.38910 (4)	0.0376 (2)	
Cl2	1.01393 (8)	0.41297 (5)	0.26911 (5)	0.0376 (2)	
Cl3	0.68442 (9)	0.47149 (4)	0.35712 (4)	0.03178 (17)	
Cl4	0.74952 (9)	0.53595 (4)	0.14312 (5)	0.03448 (18)	
Cl5	0.36171 (9)	0.50447 (5)	0.17470 (5)	0.03609 (19)	
Cl6	0.52068 (10)	0.42482 (6)	0.01852 (4)	0.0458 (2)	
O1	0.7587 (2)	0.19798 (12)	0.07832 (11)	0.0317 (5)	
O2	0.9464 (2)	0.22647 (11)	0.17462 (10)	0.0241 (4)	
O3	0.8625 (2)	0.34124 (12)	0.10127 (10)	0.0284 (5)	
P1	0.79832 (8)	0.26190 (4)	0.13825 (4)	0.02059 (16)	
Ru1	0.59449 (2)	0.290061 (12)	0.205038 (11)	0.01667 (5)	
Sn1	0.775835 (19)	0.357677 (11)	0.292932 (10)	0.02028 (4)	
Sn2	0.57536 (2)	0.427118 (11)	0.139400 (9)	0.02059 (4)	

### Atomic displacement parameters (Å^2^)

**Table:**

	*U*^11^	*U*^22^	*U*^33^	*U*^12^	*U*^13^	*U*^23^
C1	0.0156 (14)	0.0210 (15)	0.0272 (16)	−0.0021 (12)	−0.0001 (13)	−0.0017 (12)
C2	0.0201 (15)	0.0139 (14)	0.0306 (19)	−0.0004 (11)	0.0004 (13)	0.0023 (12)
C3	0.0260 (16)	0.0221 (15)	0.0224 (17)	−0.0048 (12)	0.0017 (14)	0.0062 (14)
C4	0.0224 (15)	0.0224 (16)	0.0292 (16)	−0.0051 (12)	0.0087 (14)	−0.0015 (12)
C5	0.0150 (14)	0.0216 (16)	0.0378 (17)	0.0000 (12)	0.0064 (12)	−0.0023 (13)
C6	0.0146 (14)	0.0235 (16)	0.0337 (18)	−0.0006 (12)	−0.0033 (13)	0.0025 (13)
C7	0.0255 (17)	0.0334 (17)	0.0302 (18)	0.0005 (14)	−0.0040 (14)	−0.0062 (13)
C8	0.0368 (19)	0.046 (2)	0.051 (2)	−0.0132 (16)	0.0022 (18)	−0.0195 (19)
C9	0.052 (2)	0.048 (2)	0.0291 (19)	0.0073 (18)	−0.0113 (17)	−0.0041 (15)
C10	0.0404 (19)	0.0359 (19)	0.0334 (18)	−0.0027 (15)	0.0139 (16)	−0.0040 (14)
C11	0.036 (2)	0.110 (4)	0.073 (3)	−0.001 (2)	0.016 (2)	−0.063 (3)
C12	0.085 (4)	0.072 (3)	0.041 (3)	0.031 (2)	0.001 (2)	−0.020 (2)
C21	0.0260 (17)	0.0215 (15)	0.045 (2)	0.0014 (13)	0.0013 (15)	0.0075 (13)
C22	0.0243 (17)	0.0323 (16)	0.0409 (19)	0.0047 (14)	−0.0020 (15)	0.0071 (14)
C31	0.0286 (17)	0.054 (2)	0.0482 (19)	0.0020 (18)	0.0162 (15)	0.0070 (19)
C32	0.079 (3)	0.039 (2)	0.080 (3)	0.006 (2)	0.052 (2)	0.014 (2)
Cl1	0.0505 (5)	0.0333 (5)	0.0291 (4)	0.0047 (4)	−0.0129 (4)	0.0055 (3)
Cl2	0.0212 (4)	0.0391 (5)	0.0525 (5)	−0.0038 (3)	0.0032 (3)	−0.0062 (4)
Cl3	0.0368 (4)	0.0253 (4)	0.0333 (4)	0.0046 (3)	0.0050 (4)	−0.0061 (3)
Cl4	0.0323 (4)	0.0228 (4)	0.0483 (5)	−0.0040 (3)	0.0031 (4)	−0.0032 (3)
Cl5	0.0298 (4)	0.0277 (4)	0.0508 (5)	0.0096 (3)	0.0034 (4)	0.0030 (4)
Cl6	0.0752 (6)	0.0395 (4)	0.0226 (4)	0.0001 (5)	−0.0111 (4)	0.0019 (4)
O1	0.0262 (12)	0.0389 (12)	0.0301 (12)	0.0032 (9)	0.0032 (10)	−0.0136 (9)
O2	0.0196 (11)	0.0235 (10)	0.0292 (11)	0.0023 (9)	0.0018 (8)	0.0016 (9)
O3	0.0219 (10)	0.0313 (12)	0.0319 (11)	0.0030 (9)	0.0075 (9)	0.0068 (9)
P1	0.0188 (4)	0.0223 (4)	0.0207 (4)	0.0027 (3)	0.0016 (3)	−0.0012 (3)
Ru1	0.01642 (11)	0.01575 (10)	0.01784 (11)	0.00187 (9)	0.00057 (9)	0.00037 (10)
Sn1	0.02014 (9)	0.02031 (9)	0.02040 (9)	0.00166 (9)	−0.00160 (8)	−0.00163 (10)
Sn2	0.02466 (10)	0.01701 (8)	0.02010 (9)	0.00180 (9)	−0.00106 (9)	0.00119 (9)

### Geometric parameters (Å, °)

**Table:**

C1—C6	1.406 (4)	C11—H11A	0.9700
C1—C2	1.421 (4)	C11—H11B	0.9700
C1—C7	1.515 (4)	C12—H12A	0.9600
C1—Ru1	2.310 (3)	C12—H12B	0.9600
C2—C3	1.400 (4)	C12—H12C	0.9600
C2—Ru1	2.242 (3)	C21—O2	1.462 (3)
C2—H2	0.9300	C21—C22	1.491 (4)
C3—C4	1.422 (4)	C21—H21A	0.9700
C3—Ru1	2.246 (3)	C21—H21B	0.9700
C3—H3	0.9300	C22—H22A	0.9600
C4—C5	1.398 (4)	C22—H22B	0.9600
C4—C10	1.505 (4)	C22—H22C	0.9600
C4—Ru1	2.314 (3)	C31—O3	1.461 (3)
C5—C6	1.407 (4)	C31—C32	1.471 (5)
C5—Ru1	2.260 (3)	C31—H31A	0.9700
C5—H5	0.9300	C31—H31B	0.9700
C6—Ru1	2.244 (3)	C32—H32A	0.9600
C6—H6	0.9300	C32—H32B	0.9600
C7—C9	1.513 (4)	C32—H32C	0.9600
C7—C8	1.528 (5)	Cl1—Sn1	2.3679 (8)
C7—H7	0.9800	Cl2—Sn1	2.3449 (8)
C8—H8A	0.9600	Cl3—Sn1	2.3621 (7)
C8—H8B	0.9600	Cl4—Sn2	2.3555 (8)
C8—H8C	0.9600	Cl5—Sn2	2.3761 (8)
C9—H9A	0.9600	Cl6—Sn2	2.3422 (7)
C9—H9B	0.9600	O1—P1	1.581 (2)
C9—H9C	0.9600	O2—P1	1.595 (2)
C10—H10A	0.9600	O3—P1	1.577 (2)
C10—H10B	0.9600	P1—Ru1	2.2579 (8)
C10—H10C	0.9600	Ru1—Sn2	2.5619 (3)
C11—C12	1.424 (5)	Ru1—Sn1	2.5669 (3)
C11—O1	1.454 (4)		
			
C6—C1—C2	116.5 (3)	H21A—C21—H21B	108.2
C6—C1—C7	122.9 (3)	C21—C22—H22A	109.5
C2—C1—C7	120.4 (3)	C21—C22—H22B	109.5
C6—C1—Ru1	69.48 (16)	H22A—C22—H22B	109.5
C2—C1—Ru1	69.24 (15)	C21—C22—H22C	109.5
C7—C1—Ru1	136.4 (2)	H22A—C22—H22C	109.5
C3—C2—C1	121.8 (3)	H22B—C22—H22C	109.5
C3—C2—Ru1	71.95 (15)	O3—C31—C32	108.7 (3)
C1—C2—Ru1	74.43 (16)	O3—C31—H31A	109.9
C3—C2—H2	119.1	C32—C31—H31A	109.9
C1—C2—H2	119.1	O3—C31—H31B	109.9
Ru1—C2—H2	126.5	C32—C31—H31B	109.9
C2—C3—C4	120.7 (3)	H31A—C31—H31B	108.3
C2—C3—Ru1	71.70 (15)	C31—C32—H32A	109.5
C4—C3—Ru1	74.46 (16)	C31—C32—H32B	109.5
C2—C3—H3	119.7	H32A—C32—H32B	109.5
C4—C3—H3	119.7	C31—C32—H32C	109.5
Ru1—C3—H3	126.0	H32A—C32—H32C	109.5
C5—C4—C3	117.6 (3)	H32B—C32—H32C	109.5
C5—C4—C10	121.0 (3)	C11—O1—P1	124.1 (2)
C3—C4—C10	121.5 (3)	C21—O2—P1	119.14 (17)
C5—C4—Ru1	70.11 (15)	C31—O3—P1	129.4 (2)
C3—C4—Ru1	69.24 (15)	O3—P1—O1	107.53 (12)
C10—C4—Ru1	133.19 (19)	O3—P1—O2	100.96 (10)
C4—C5—C6	121.3 (3)	O1—P1—O2	104.84 (11)
C4—C5—Ru1	74.31 (16)	O3—P1—Ru1	111.89 (8)
C6—C5—Ru1	71.18 (16)	O1—P1—Ru1	110.96 (8)
C4—C5—H5	119.4	O2—P1—Ru1	119.63 (8)
C6—C5—H5	119.4	C2—Ru1—C6	64.79 (10)
Ru1—C5—H5	127.2	C2—Ru1—C3	36.35 (9)
C1—C6—C5	121.9 (3)	C6—Ru1—C3	76.80 (11)
C1—C6—Ru1	74.60 (17)	C2—Ru1—P1	96.78 (8)
C5—C6—Ru1	72.41 (16)	C6—Ru1—P1	123.48 (8)
C1—C6—H6	119.1	C3—Ru1—P1	120.14 (8)
C5—C6—H6	119.1	C2—Ru1—C5	76.43 (10)
Ru1—C6—H6	125.8	C6—Ru1—C5	36.41 (10)
C9—C7—C1	114.5 (3)	C3—Ru1—C5	64.73 (10)
C9—C7—C8	111.3 (3)	P1—Ru1—C5	159.80 (8)
C1—C7—C8	106.9 (3)	C2—Ru1—C1	36.34 (10)
C9—C7—H7	108.0	C6—Ru1—C1	35.93 (10)
C1—C7—H7	108.0	C3—Ru1—C1	65.51 (11)
C8—C7—H7	108.0	P1—Ru1—C1	98.08 (7)
C7—C8—H8A	109.5	C5—Ru1—C1	65.09 (10)
C7—C8—H8B	109.5	C2—Ru1—C4	65.10 (10)
H8A—C8—H8B	109.5	C6—Ru1—C4	64.86 (10)
C7—C8—H8C	109.5	C3—Ru1—C4	36.30 (10)
H8A—C8—H8C	109.5	P1—Ru1—C4	155.96 (7)
H8B—C8—H8C	109.5	C5—Ru1—C4	35.58 (10)
C7—C9—H9A	109.5	C1—Ru1—C4	76.90 (10)
C7—C9—H9B	109.5	C2—Ru1—Sn2	150.01 (8)
H9A—C9—H9B	109.5	C6—Ru1—Sn2	88.03 (7)
C7—C9—H9C	109.5	C3—Ru1—Sn2	152.26 (7)
H9A—C9—H9C	109.5	P1—Ru1—Sn2	87.608 (19)
H9B—C9—H9C	109.5	C5—Ru1—Sn2	89.28 (7)
C4—C10—H10A	109.5	C1—Ru1—Sn2	113.70 (7)
C4—C10—H10B	109.5	C4—Ru1—Sn2	116.07 (6)
H10A—C10—H10B	109.5	C2—Ru1—Sn1	120.77 (8)
C4—C10—H10C	109.5	C6—Ru1—Sn1	149.24 (8)
H10A—C10—H10C	109.5	C3—Ru1—Sn1	92.35 (8)
H10B—C10—H10C	109.5	P1—Ru1—Sn1	86.94 (2)
C12—C11—O1	111.4 (3)	C5—Ru1—Sn1	112.96 (8)
C12—C11—H11A	109.3	C1—Ru1—Sn1	156.83 (7)
O1—C11—H11A	109.3	C4—Ru1—Sn1	89.29 (7)
C12—C11—H11B	109.3	Sn2—Ru1—Sn1	89.009 (9)
O1—C11—H11B	109.3	Cl2—Sn1—Cl3	96.22 (3)
H11A—C11—H11B	108.0	Cl2—Sn1—Cl1	98.76 (3)
C11—C12—H12A	109.5	Cl3—Sn1—Cl1	98.67 (3)
C11—C12—H12B	109.5	Cl2—Sn1—Ru1	127.39 (2)
H12A—C12—H12B	109.5	Cl3—Sn1—Ru1	117.06 (2)
C11—C12—H12C	109.5	Cl1—Sn1—Ru1	113.66 (2)
H12A—C12—H12C	109.5	Cl6—Sn2—Cl4	100.24 (3)
H12B—C12—H12C	109.5	Cl6—Sn2—Cl5	96.77 (3)
O2—C21—C22	109.8 (2)	Cl4—Sn2—Cl5	96.78 (3)
O2—C21—H21A	109.7	Cl6—Sn2—Ru1	118.34 (3)
C22—C21—H21A	109.7	Cl4—Sn2—Ru1	126.73 (2)
O2—C21—H21B	109.7	Cl5—Sn2—Ru1	112.26 (2)
C22—C21—H21B	109.7		
			
C6—C1—C2—C3	3.9 (4)	O1—P1—Ru1—Sn1	166.88 (9)
C7—C1—C2—C3	−171.0 (3)	O2—P1—Ru1—Sn1	44.64 (9)
Ru1—C1—C2—C3	56.5 (2)	C4—C5—Ru1—C2	65.99 (16)
C6—C1—C2—Ru1	−52.6 (2)	C6—C5—Ru1—C2	−65.73 (17)
C7—C1—C2—Ru1	132.5 (3)	C4—C5—Ru1—C6	131.7 (2)
C1—C2—C3—C4	0.7 (4)	C4—C5—Ru1—C3	29.33 (15)
Ru1—C2—C3—C4	58.4 (2)	C6—C5—Ru1—C3	−102.39 (18)
C1—C2—C3—Ru1	−57.7 (3)	C4—C5—Ru1—P1	138.28 (19)
C2—C3—C4—C5	−4.5 (4)	C6—C5—Ru1—P1	6.6 (3)
Ru1—C3—C4—C5	52.5 (2)	C4—C5—Ru1—C1	102.70 (17)
C2—C3—C4—C10	174.2 (3)	C6—C5—Ru1—C1	−29.02 (16)
Ru1—C3—C4—C10	−128.8 (3)	C6—C5—Ru1—C4	−131.7 (2)
C2—C3—C4—Ru1	−57.0 (2)	C4—C5—Ru1—Sn2	−140.62 (15)
C3—C4—C5—C6	3.6 (4)	C6—C5—Ru1—Sn2	87.66 (16)
C10—C4—C5—C6	−175.1 (3)	C4—C5—Ru1—Sn1	−52.00 (16)
Ru1—C4—C5—C6	55.8 (2)	C6—C5—Ru1—Sn1	176.28 (14)
C3—C4—C5—Ru1	−52.1 (2)	C6—C1—Ru1—C2	130.6 (3)
C10—C4—C5—Ru1	129.2 (2)	C7—C1—Ru1—C2	−112.7 (4)
C2—C1—C6—C5	−4.8 (4)	C2—C1—Ru1—C6	−130.6 (3)
C7—C1—C6—C5	170.0 (3)	C7—C1—Ru1—C6	116.7 (4)
Ru1—C1—C6—C5	−57.3 (2)	C6—C1—Ru1—C3	101.59 (19)
C2—C1—C6—Ru1	52.5 (2)	C2—C1—Ru1—C3	−29.03 (17)
C7—C1—C6—Ru1	−132.7 (3)	C7—C1—Ru1—C3	−141.7 (3)
C4—C5—C6—C1	1.1 (4)	C6—C1—Ru1—P1	−138.91 (17)
Ru1—C5—C6—C1	58.3 (3)	C2—C1—Ru1—P1	90.47 (17)
C4—C5—C6—Ru1	−57.2 (2)	C7—C1—Ru1—P1	−22.2 (3)
C6—C1—C7—C9	30.2 (4)	C6—C1—Ru1—C5	29.39 (17)
C2—C1—C7—C9	−155.2 (3)	C2—C1—Ru1—C5	−101.24 (19)
Ru1—C1—C7—C9	−64.1 (4)	C7—C1—Ru1—C5	146.1 (3)
C6—C1—C7—C8	−93.6 (4)	C6—C1—Ru1—C4	65.03 (18)
C2—C1—C7—C8	81.0 (3)	C2—C1—Ru1—C4	−65.59 (18)
Ru1—C1—C7—C8	172.1 (2)	C7—C1—Ru1—C4	−178.3 (3)
C12—C11—O1—P1	−172.8 (3)	C6—C1—Ru1—Sn2	−47.97 (18)
C22—C21—O2—P1	−161.1 (2)	C2—C1—Ru1—Sn2	−178.59 (15)
C32—C31—O3—P1	151.6 (3)	C7—C1—Ru1—Sn2	68.7 (3)
C31—O3—P1—O1	−80.5 (3)	C6—C1—Ru1—Sn1	119.9 (2)
C31—O3—P1—O2	29.0 (3)	C2—C1—Ru1—Sn1	−10.8 (3)
C31—O3—P1—Ru1	157.4 (2)	C7—C1—Ru1—Sn1	−123.5 (3)
C11—O1—P1—O3	57.4 (3)	C5—C4—Ru1—C2	−101.78 (18)
C11—O1—P1—O2	−49.4 (3)	C3—C4—Ru1—C2	29.78 (17)
C11—O1—P1—Ru1	−179.9 (3)	C10—C4—Ru1—C2	144.0 (3)
C21—O2—P1—O3	178.3 (2)	C5—C4—Ru1—C6	−29.30 (16)
C21—O2—P1—O1	−70.1 (2)	C3—C4—Ru1—C6	102.27 (18)
C21—O2—P1—Ru1	55.1 (2)	C10—C4—Ru1—C6	−143.5 (3)
C3—C2—Ru1—C6	−102.3 (2)	C5—C4—Ru1—C3	−131.6 (2)
C1—C2—Ru1—C6	29.49 (17)	C10—C4—Ru1—C3	114.2 (4)
C1—C2—Ru1—C3	131.8 (3)	C5—C4—Ru1—P1	−145.66 (16)
C3—C2—Ru1—P1	133.74 (17)	C3—C4—Ru1—P1	−14.1 (3)
C1—C2—Ru1—P1	−94.43 (16)	C10—C4—Ru1—P1	100.1 (3)
C3—C2—Ru1—C5	−65.61 (19)	C3—C4—Ru1—C5	131.6 (2)
C1—C2—Ru1—C5	66.22 (18)	C10—C4—Ru1—C5	−114.2 (3)
C3—C2—Ru1—C1	−131.8 (3)	C5—C4—Ru1—C1	−65.28 (16)
C3—C2—Ru1—C4	−29.75 (18)	C3—C4—Ru1—C1	66.29 (17)
C1—C2—Ru1—C4	102.09 (19)	C10—C4—Ru1—C1	−179.5 (3)
C3—C2—Ru1—Sn2	−129.25 (16)	C5—C4—Ru1—Sn2	44.93 (17)
C1—C2—Ru1—Sn2	2.6 (3)	C3—C4—Ru1—Sn2	176.50 (14)
C3—C2—Ru1—Sn1	43.3 (2)	C10—C4—Ru1—Sn2	−69.3 (3)
C1—C2—Ru1—Sn1	175.09 (14)	C5—C4—Ru1—Sn1	133.48 (15)
C1—C6—Ru1—C2	−29.81 (17)	C3—C4—Ru1—Sn1	−94.95 (16)
C5—C6—Ru1—C2	101.62 (19)	C10—C4—Ru1—Sn1	19.2 (3)
C1—C6—Ru1—C3	−66.31 (18)	C2—Ru1—Sn1—Cl2	120.51 (9)
C5—C6—Ru1—C3	65.12 (17)	C6—Ru1—Sn1—Cl2	−147.70 (15)
C1—C6—Ru1—P1	51.28 (19)	C3—Ru1—Sn1—Cl2	144.50 (8)
C5—C6—Ru1—P1	−177.29 (13)	P1—Ru1—Sn1—Cl2	24.44 (3)
C1—C6—Ru1—C5	−131.4 (3)	C5—Ru1—Sn1—Cl2	−152.02 (8)
C5—C6—Ru1—C1	131.4 (3)	C1—Ru1—Sn1—Cl2	127.92 (18)
C1—C6—Ru1—C4	−102.77 (19)	C4—Ru1—Sn1—Cl2	−179.31 (7)
C5—C6—Ru1—C4	28.66 (16)	Sn2—Ru1—Sn1—Cl2	−63.22 (3)
C1—C6—Ru1—Sn2	137.11 (17)	C2—Ru1—Sn1—Cl3	−116.57 (9)
C5—C6—Ru1—Sn2	−91.46 (16)	C6—Ru1—Sn1—Cl3	−24.78 (15)
C1—C6—Ru1—Sn1	−138.14 (15)	C3—Ru1—Sn1—Cl3	−92.58 (8)
C5—C6—Ru1—Sn1	−6.7 (3)	P1—Ru1—Sn1—Cl3	147.36 (3)
C4—C3—Ru1—C2	−130.5 (3)	C5—Ru1—Sn1—Cl3	−29.10 (8)
C2—C3—Ru1—C6	65.21 (19)	C1—Ru1—Sn1—Cl3	−109.16 (18)
C4—C3—Ru1—C6	−65.32 (17)	C4—Ru1—Sn1—Cl3	−56.39 (7)
C2—C3—Ru1—P1	−56.1 (2)	Sn2—Ru1—Sn1—Cl3	59.70 (2)
C4—C3—Ru1—P1	173.41 (14)	C2—Ru1—Sn1—Cl1	−2.45 (9)
C2—C3—Ru1—C5	101.8 (2)	C6—Ru1—Sn1—Cl1	89.34 (15)
C4—C3—Ru1—C5	−28.77 (16)	C3—Ru1—Sn1—Cl1	21.54 (8)
C2—C3—Ru1—C1	29.02 (18)	P1—Ru1—Sn1—Cl1	−98.52 (3)
C4—C3—Ru1—C1	−101.51 (18)	C5—Ru1—Sn1—Cl1	85.02 (8)
C2—C3—Ru1—C4	130.5 (3)	C1—Ru1—Sn1—Cl1	4.96 (18)
C2—C3—Ru1—Sn2	123.76 (18)	C4—Ru1—Sn1—Cl1	57.73 (7)
C4—C3—Ru1—Sn2	−6.8 (3)	Sn2—Ru1—Sn1—Cl1	173.82 (2)
C2—C3—Ru1—Sn1	−143.89 (17)	C2—Ru1—Sn2—Cl6	−33.98 (16)
C4—C3—Ru1—Sn1	85.58 (16)	C6—Ru1—Sn2—Cl6	−58.17 (8)
O3—P1—Ru1—C2	166.35 (11)	C3—Ru1—Sn2—Cl6	−114.37 (17)
O1—P1—Ru1—C2	46.25 (12)	P1—Ru1—Sn2—Cl6	65.47 (3)
O2—P1—Ru1—C2	−75.99 (11)	C5—Ru1—Sn2—Cl6	−94.56 (8)
O3—P1—Ru1—C6	102.18 (12)	C1—Ru1—Sn2—Cl6	−32.31 (8)
O1—P1—Ru1—C6	−17.93 (13)	C4—Ru1—Sn2—Cl6	−118.83 (8)
O2—P1—Ru1—C6	−140.17 (12)	Sn1—Ru1—Sn2—Cl6	152.45 (3)
O3—P1—Ru1—C3	−163.96 (12)	C2—Ru1—Sn2—Cl4	−164.87 (15)
O1—P1—Ru1—C3	75.93 (13)	C6—Ru1—Sn2—Cl4	170.95 (8)
O2—P1—Ru1—C3	−46.31 (13)	C3—Ru1—Sn2—Cl4	114.74 (17)
O3—P1—Ru1—C5	97.5 (2)	P1—Ru1—Sn2—Cl4	−65.42 (3)
O1—P1—Ru1—C5	−22.6 (2)	C5—Ru1—Sn2—Cl4	134.55 (8)
O2—P1—Ru1—C5	−144.8 (2)	C1—Ru1—Sn2—Cl4	−163.20 (8)
O3—P1—Ru1—C1	129.72 (11)	C4—Ru1—Sn2—Cl4	110.28 (8)
O1—P1—Ru1—C1	9.61 (12)	Sn1—Ru1—Sn2—Cl4	21.56 (3)
O2—P1—Ru1—C1	−112.63 (11)	C2—Ru1—Sn2—Cl5	77.46 (15)
O3—P1—Ru1—C4	−154.36 (19)	C6—Ru1—Sn2—Cl5	53.27 (8)
O1—P1—Ru1—C4	85.5 (2)	C3—Ru1—Sn2—Cl5	−2.94 (17)
O2—P1—Ru1—C4	−36.7 (2)	P1—Ru1—Sn2—Cl5	176.91 (3)
O3—P1—Ru1—Sn2	16.12 (8)	C5—Ru1—Sn2—Cl5	16.87 (8)
O1—P1—Ru1—Sn2	−103.99 (9)	C1—Ru1—Sn2—Cl5	79.13 (8)
O2—P1—Ru1—Sn2	133.78 (9)	C4—Ru1—Sn2—Cl5	−7.39 (8)
O3—P1—Ru1—Sn1	−73.01 (9)	Sn1—Ru1—Sn2—Cl5	−96.11 (2)
